# After the Operation

**Published:** 1992-03

**Authors:** M. D. Read


M. D. Read

### AFTER THE OPERATION

Over the past couple of years attention has been increasingly focused on greater use of day surgery. It has been estimated that up to 50% of surgical cases could potentially be treated as day cases. At the same time we have seen the emergence of "minimally invasive surgery". Essentially based on laparoscopic techniques, this rapidly developing concept has certainly caught the imagination and an increasing number of operations are being performed via the laparoscope, either as day cases or with an overnight stay. Operations include cholescystectomy, appendicectomy and hernia repair, oophorectomy, salpingectomy and even hysterectomy and nephrectomy. We now have Associations for those with a particular interest in day surgery and minimal invasive surgery.

The advantages of these techniques to the patient are obvious, with a much shorter stay in hospital and, where minimal invasive techniques are used, there are smaller scars. The benefits to the hospital are rather less clear cut. Certainly there is potentially a lesser need for nursing staff at night and weekends but day surgery techniques demand a high level of expertise from both surgeon and anaesthetist and, unless one is going to appoint additional staff, it is not going to make a major impact on the waiting list, as one is merely transferring the patient from one operating list to another. The "minimally invasive" techniques demand a much greater level of expertise and after the initial explosion of interest and "band wagon" effect, we are now beginning to see notes of caution sounded as the complications become apparent (Observer — Sunday 22 September 1991). One also hears increasingly of complications which Gynaecologists were experiencing 20 years ago when laparoscopy was introduced into that speciality and which prompted the report published by the Royal College of Obstetricians and Gynaecologists in 1977. There is no doubt that laparoscopic surgical techniques in general take much longer than the traditional laparotomy procedures, thus far less operating can be performed on each operating list. This is likely to have an adverse effect on waiting lists.

During the time these developments have been occurring, we have heard little of the "traditional" surgical techniques for these operations which are now being offered laparoscopically. They still represent the majority of day to day surgery and will probably continue to do so. There may have been a slight reduction in the length of post operative stay but, in general, laparotomies are associated with a significant post operative inpatient stay. Is this really necessary? When one considers the results of laparoscopic surgical techniques, one really must question our traditional pattern of practice. If one can remove an appendix through the laparoscope with two or three 1cm incisions and allow the patient home the same day, why is it necessary for the patient to stay up to 3 or even 4 days when they have a 3cm incision? The total scar size is probably the same, the surgery to the bowel is certainly the same and the anaesthetic time is considerably shorter with the laparotomy technique!

"Laparoscopic" hysterectomies are now being performed and the operation currently takes up to two hours. The patients suitable for this procedure are those with a normal sized uterus with no intrapelvic adhesions or fibrosis. The time for a laparotomy procedure for such a case would be around 30 minutes. Why then does a women, with three or four 1 cm incisions and 2 hours under anaesthesia go home the next day when a women who has one 5cm incision and a 30 minute anaesthetic need to stay in for several days. (Recent media articles about laparoscopic techniques have suggested that the average stay for conventional hysterectomies may be as much as 8 days!). After all, the same pedicles have to be ligated, the same vessels secured and the uterus has to be removed, whichever way it is performed.

If we therefore have a critical appraisal of our current practice (dare one say audit?) there may well be scope for considerable changes in the present pattern of practice. With appropriate explanation to the patient or the patient's family and carers pre-operatively about what can be realistically **expected** post-operatively, a positive approach would be achieved and the ingrained old wives' tales gradually abolished.

Patients who go home the day after ''minimally invasive'' procedures are up and about much more quickly and go to work much more quickly than those having a similar procedure performed by traditional laparotomy methods. Why? The 'internal' part of the operation is essentially the same. The only difference is the abdominal incision and if we are considering elective procedures performed on fit patients the fact that early return to ''normality'' is possible, then why are we not encouraging it following traditional procedures?

It seems that this situation has arisen from a combination of ''surgical tradition'' and patients' expectations and anticipations. Ladies coming in to hospital for a hysterctomy have received all the ''community'' advice and **expect** to stay a week or more and to be a virtual invalid for several weeks if not months after the procedure. That this is patently not the case was dramatically illustrated recently when a wonderful patient of mine in her early 70's required hysterectomy for an endometrial carcinoma. She had her operation on the Friday and it was all we could do to keep her in hospital until the Monday. On the Wednesday I received a phone call from the GP saying he could swear he had seen her coming out of the Supermarket and going into the Bookies! He was quite right. She couldn't be bothered with being an invalid as she had far more important things to attend to.

There is no doubt that where an elective procedure is uncomplicated, the post operative recovery is largely supratentorial and will depend on the individual's expectations and personality.

M. D. Read

### OVERHEARD CONVERSATION

**Q. What is a performance-related bonus!**

A. This is a cash incentive offered to hospital managers as a reward for treating more patients.

**Q. But hospital managers don't treat patients, do they?**

A. Not yet.

**Q. Who does?**

A. Mainly doctors and nurses.

**Q. Are they given a performance-related bonus?**

A. No.

**Q. Odd system, isn't it?**

A. Yes.

Anon.

